# Supplementary material for: Modeling glioblastoma heterogeneity as a dynamic network of cell states
Source: Mol Syst Biol. 2021 Sep 16;17(9):e10105. doi: 10.15252/msb.202010105 (PMC8444284; doi:10.15252/msb.202010105)
Supplement: Supplementary file 5 — Source Data for Figure 3 [file MSB-17-e10105-s001.zip › Figure3A_sourcedata/GSEA_3065/nosigdb_state5.GseaPreranked.1622718435914/NB_KI_CLUSTER2.html]

Details for gene set NB\_KI\_CLUSTER2[GSEA]

|  || Dataset | state5 |
| Phenotype | NoPhenotypeAvailable |
| Upregulated in class | na\_pos |
| GeneSet | NB\_KI\_CLUSTER2 |
| Enrichment Score (ES) | 0.40610236 |
| Normalized Enrichment Score (NES) | 1.8412633 |
| Nominal p-value | 0.0 |
| FDR q-value | 0.0069141397 |
| FWER p-Value | 0.054 |
Table: GSEA Results Summary

  

Fig 1: Enrichment plot: NB\_KI\_CLUSTER2      
 Profile of the Running ES Score & Positions of GeneSet Members on the Rank Ordered List

  

| PROBE | GENE SYMBOL | GENE\_TITLE | RANK IN GENE LIST | RANK METRIC SCORE | RUNNING ES | CORE ENRICHMENT || 1 | KCNQ1OT1 |  |  | 8 | 0.931 | 0.0199 | Yes |
| 2 | KANSL1 |  |  | 34 | 0.721 | 0.0332 | Yes |
| 3 | PRH1 |  |  | 35 | 0.718 | 0.0493 | Yes |
| 4 | ARGLU1 |  |  | 50 | 0.642 | 0.0621 | Yes |
| 5 | MARCH6 |  |  | 90 | 0.543 | 0.0698 | Yes |
| 6 | LAPTM4B |  |  | 97 | 0.532 | 0.0811 | Yes |
| 7 | DSEL |  |  | 107 | 0.518 | 0.0916 | Yes |
| 8 | MTMR4 |  |  | 108 | 0.518 | 0.1032 | Yes |
| 9 | EFHC1 |  |  | 148 | 0.486 | 0.1096 | Yes |
| 10 | PNISR |  |  | 150 | 0.482 | 0.1203 | Yes |
| 11 | DNM1 |  |  | 152 | 0.480 | 0.1310 | Yes |
| 12 | CLSTN1 |  |  | 154 | 0.478 | 0.1416 | Yes |
| 13 | DYNC1H1 |  |  | 160 | 0.475 | 0.1517 | Yes |
| 14 | CLU |  |  | 162 | 0.474 | 0.1622 | Yes |
| 15 | CHST12 |  |  | 206 | 0.436 | 0.1670 | Yes |
| 16 | RTN4 |  |  | 221 | 0.425 | 0.1749 | Yes |
| 17 | ALCAM |  |  | 225 | 0.422 | 0.1840 | Yes |
| 18 | MUM1 |  |  | 263 | 0.401 | 0.1888 | Yes |
| 19 | ACAP3 |  |  | 267 | 0.399 | 0.1974 | Yes |
| 20 | SLC39A6 |  |  | 269 | 0.399 | 0.2062 | Yes |
| 21 | TANC2 |  |  | 273 | 0.398 | 0.2148 | Yes |
| 22 | CHD9 |  |  | 275 | 0.396 | 0.2236 | Yes |
| 23 | PCDH9 |  |  | 285 | 0.390 | 0.2313 | Yes |
| 24 | FAM215B |  |  | 302 | 0.381 | 0.2380 | Yes |
| 25 | NHLRC3 |  |  | 318 | 0.374 | 0.2446 | Yes |
| 26 | NRP2 |  |  | 319 | 0.374 | 0.2530 | Yes |
| 27 | KIDINS220 |  |  | 325 | 0.372 | 0.2608 | Yes |
| 28 | CLCN3 |  |  | 356 | 0.360 | 0.2654 | Yes |
| 29 | MAPK10 |  |  | 376 | 0.350 | 0.2711 | Yes |
| 30 | RETREG1 |  |  | 403 | 0.340 | 0.2757 | Yes |
| 31 | AUTS2 |  |  | 434 | 0.330 | 0.2797 | Yes |
| 32 | SRGAP3 |  |  | 446 | 0.326 | 0.2857 | Yes |
| 33 | SLC39A10 |  |  | 448 | 0.326 | 0.2929 | Yes |
| 34 | SLC25A36 |  |  | 482 | 0.317 | 0.2963 | Yes |
| 35 | MSI2 |  |  | 496 | 0.313 | 0.3018 | Yes |
| 36 | VEZT |  |  | 510 | 0.308 | 0.3072 | Yes |
| 37 | ZNF397 |  |  | 529 | 0.303 | 0.3119 | Yes |
| 38 | NPDC1 |  |  | 581 | 0.290 | 0.3126 | Yes |
| 39 | BICD1 |  |  | 586 | 0.289 | 0.3186 | Yes |
| 40 | EXOC4 |  |  | 592 | 0.287 | 0.3244 | Yes |
| 41 | TTLL7 |  |  | 602 | 0.285 | 0.3298 | Yes |
| 42 | AHI1 |  |  | 609 | 0.283 | 0.3354 | Yes |
| 43 | RSBN1L |  |  | 650 | 0.275 | 0.3370 | Yes |
| 44 | ZNF292 |  |  | 656 | 0.274 | 0.3426 | Yes |
| 45 | ZKSCAN1 |  |  | 678 | 0.269 | 0.3462 | Yes |
| 46 | TUT4 |  |  | 691 | 0.266 | 0.3508 | Yes |
| 47 | GOLM1 |  |  | 692 | 0.265 | 0.3568 | Yes |
| 48 | TSC2 |  |  | 703 | 0.261 | 0.3615 | Yes |
| 49 | CDK5RAP3 |  |  | 823 | 0.237 | 0.3531 | Yes |
| 50 | PTPRA |  |  | 855 | 0.232 | 0.3548 | Yes |
| 51 | ZNF37A |  |  | 870 | 0.230 | 0.3583 | Yes |
| 52 | WDR6 |  |  | 903 | 0.225 | 0.3597 | Yes |
| 53 | PTBP2 |  |  | 914 | 0.222 | 0.3636 | Yes |
| 54 | SORBS2 |  |  | 925 | 0.221 | 0.3674 | Yes |
| 55 | SRGAP1 |  |  | 937 | 0.220 | 0.3710 | Yes |
| 56 | BAZ2B |  |  | 974 | 0.214 | 0.3717 | Yes |
| 57 | ZDHHC21 |  |  | 984 | 0.212 | 0.3754 | Yes |
| 58 | CASC15 |  |  | 1017 | 0.208 | 0.3764 | Yes |
| 59 | AKAP9 |  |  | 1021 | 0.208 | 0.3807 | Yes |
| 60 | TMX4 |  |  | 1025 | 0.208 | 0.3851 | Yes |
| 61 | ANKRD12 |  |  | 1083 | 0.200 | 0.3830 | Yes |
| 62 | ICA1L |  |  | 1124 | 0.195 | 0.3828 | Yes |
| 63 | PWAR6 |  |  | 1133 | 0.194 | 0.3863 | Yes |
| 64 | CSNK1E |  |  | 1134 | 0.194 | 0.3906 | Yes |
| 65 | EIF4A2 |  |  | 1153 | 0.191 | 0.3928 | Yes |
| 66 | RBM26 |  |  | 1154 | 0.191 | 0.3971 | Yes |
| 67 | RNMT |  |  | 1163 | 0.190 | 0.4004 | Yes |
| 68 | PRRC2B |  |  | 1241 | 0.180 | 0.3957 | Yes |
| 69 | MAFG |  |  | 1245 | 0.180 | 0.3993 | Yes |
| 70 | ZNF91 |  |  | 1246 | 0.180 | 0.4034 | Yes |
| 71 | CCDC82 |  |  | 1258 | 0.178 | 0.4061 | Yes |
| 72 | TRIM13 |  |  | 1296 | 0.172 | 0.4057 | No |
| 73 | YPEL1 |  |  | 1363 | 0.165 | 0.4019 | No |
| 74 | HEXD |  |  | 1366 | 0.165 | 0.4053 | No |
| 75 | CASD1 |  |  | 1424 | 0.160 | 0.4024 | No |
| 76 | EPC1 |  |  | 1443 | 0.158 | 0.4038 | No |
| 77 | CLASP2 |  |  | 1504 | 0.152 | 0.4004 | No |
| 78 | PLD3 |  |  | 1562 | 0.145 | 0.3971 | No |
| 79 | SPAG9 |  |  | 1566 | 0.145 | 0.4000 | No |
| 80 | LNPK |  |  | 1572 | 0.143 | 0.4026 | No |
| 81 | PLEKHA8 |  |  | 1615 | 0.140 | 0.4010 | No |
| 82 | IP6K2 |  |  | 1621 | 0.140 | 0.4036 | No |
| 83 | CCDC88A |  |  | 1656 | 0.137 | 0.4027 | No |
| 84 | EPN2 |  |  | 1688 | 0.134 | 0.4022 | No |
| 85 | APC |  |  | 1704 | 0.133 | 0.4035 | No |
| 86 | UBN2 |  |  | 1780 | 0.128 | 0.3977 | No |
| 87 | TUBGCP4 |  |  | 1860 | 0.122 | 0.3914 | No |
| 88 | MCF2L |  |  | 1915 | 0.117 | 0.3879 | No |
| 89 | PRRT2 |  |  | 1951 | 0.115 | 0.3864 | No |
| 90 | MAP1A |  |  | 1958 | 0.114 | 0.3883 | No |
| 91 | TCF4 |  |  | 2008 | 0.111 | 0.3852 | No |
| 92 | TET1 |  |  | 2015 | 0.110 | 0.3870 | No |
| 93 | TNRC6C |  |  | 2029 | 0.110 | 0.3879 | No |
| 94 | ATP6V1H |  |  | 2034 | 0.109 | 0.3899 | No |
| 95 | MEST |  |  | 2059 | 0.108 | 0.3896 | No |
| 96 | ARHGAP21 |  |  | 2067 | 0.107 | 0.3912 | No |
| 97 | DAAM1 |  |  | 2069 | 0.107 | 0.3935 | No |
| 98 | CCZ1 |  |  | 2080 | 0.106 | 0.3947 | No |
| 99 | DNMT3A |  |  | 2133 | 0.103 | 0.3911 | No |
| 100 | CTTNBP2NL |  |  | 2180 | 0.100 | 0.3881 | No |
| 101 | PCNX2 |  |  | 2190 | 0.099 | 0.3892 | No |
| 102 | TMCO3 |  |  | 2272 | 0.093 | 0.3821 | No |
| 103 | ZFHX3 |  |  | 2308 | 0.091 | 0.3801 | No |
| 104 | EFNB2 |  |  | 2311 | 0.091 | 0.3819 | No |
| 105 | PGM2L1 |  |  | 2358 | 0.088 | 0.3786 | No |
| 106 | TTL |  |  | 2371 | 0.088 | 0.3792 | No |
| 107 | PHIP |  |  | 2406 | 0.085 | 0.3772 | No |
| 108 | LPIN1 |  |  | 2453 | 0.082 | 0.3738 | No |
| 109 | TSHZ2 |  |  | 2499 | 0.079 | 0.3704 | No |
| 110 | CPEB4 |  |  | 2511 | 0.078 | 0.3709 | No |
| 111 | SESTD1 |  |  | 2520 | 0.078 | 0.3717 | No |
| 112 | LPCAT4 |  |  | 2528 | 0.077 | 0.3727 | No |
| 113 | ABR |  |  | 2597 | 0.074 | 0.3665 | No |
| 114 | SECISBP2 |  |  | 2605 | 0.073 | 0.3674 | No |
| 115 | BPTF |  |  | 2639 | 0.071 | 0.3652 | No |
| 116 | SUPT20H |  |  | 2735 | 0.066 | 0.3558 | No |
| 117 | TRIM16 |  |  | 2768 | 0.064 | 0.3536 | No |
| 118 | PSD3 |  |  | 2778 | 0.064 | 0.3540 | No |
| 119 | SFSWAP |  |  | 2806 | 0.062 | 0.3523 | No |
| 120 | MCC |  |  | 2841 | 0.061 | 0.3498 | No |
| 121 | MYCBP2 |  |  | 2925 | 0.056 | 0.3415 | No |
| 122 | TLE4 |  |  | 2935 | 0.056 | 0.3417 | No |
| 123 | RUNDC1 |  |  | 3128 | 0.046 | 0.3208 | No |
| 124 | PRXL2A |  |  | 3169 | 0.043 | 0.3172 | No |
| 125 | LRRC37B |  |  | 3178 | 0.042 | 0.3172 | No |
| 126 | RAB3B |  |  | 3233 | 0.039 | 0.3119 | No |
| 127 | MAP4K5 |  |  | 3258 | 0.038 | 0.3100 | No |
| 128 | ZNF33A |  |  | 3326 | 0.035 | 0.3031 | No |
| 129 | ZNF429 |  |  | 3354 | 0.034 | 0.3008 | No |
| 130 | PARP6 |  |  | 3358 | 0.033 | 0.3012 | No |
| 131 | FAM171B |  |  | 3411 | 0.031 | 0.2959 | No |
| 132 | CACNB3 |  |  | 3510 | 0.027 | 0.2853 | No |
| 133 | KIF1B |  |  | 3759 | 0.017 | 0.2573 | No |
| 134 | MXI1 |  |  | 3791 | 0.016 | 0.2541 | No |
| 135 | ANK3 |  |  | 3818 | 0.015 | 0.2515 | No |
| 136 | TLN2 |  |  | 3842 | 0.014 | 0.2491 | No |
| 137 | NPEPPS |  |  | 3869 | 0.013 | 0.2464 | No |
| 138 | TSPYL4 |  |  | 3905 | 0.012 | 0.2427 | No |
| 139 | ATP6V1B2 |  |  | 3924 | 0.010 | 0.2409 | No |
| 140 | ATRX |  |  | 3933 | 0.010 | 0.2402 | No |
| 141 | DIXDC1 |  |  | 4082 | 0.004 | 0.2233 | No |
| 142 | DTX3 |  |  | 4150 | 0.001 | 0.2157 | No |
| 143 | CD200 |  |  | 4161 | 0.000 | 0.2145 | No |
| 144 | PREPL |  |  | 4184 | -0.001 | 0.2120 | No |
| 145 | GALNT11 |  |  | 4297 | -0.006 | 0.1993 | No |
| 146 | GSE1 |  |  | 4302 | -0.006 | 0.1990 | No |
| 147 | PPP1R9A |  |  | 4335 | -0.007 | 0.1955 | No |
| 148 | SMARCD3 |  |  | 4367 | -0.009 | 0.1921 | No |
| 149 | ORAI2 |  |  | 4428 | -0.011 | 0.1855 | No |
| 150 | KIAA0232 |  |  | 4475 | -0.013 | 0.1805 | No |
| 151 | FSD1 |  |  | 4477 | -0.013 | 0.1807 | No |
| 152 | DYNC1I2 |  |  | 4485 | -0.013 | 0.1802 | No |
| 153 | DBN1 |  |  | 4498 | -0.014 | 0.1792 | No |
| 154 | CEP290 |  |  | 4503 | -0.014 | 0.1790 | No |
| 155 | RHBDD2 |  |  | 4523 | -0.015 | 0.1772 | No |
| 156 | PNMA1 |  |  | 4610 | -0.019 | 0.1678 | No |
| 157 | FOXN3 |  |  | 4639 | -0.020 | 0.1650 | No |
| 158 | GNAI1 |  |  | 4664 | -0.021 | 0.1627 | No |
| 159 | DYNC1LI2 |  |  | 4674 | -0.021 | 0.1621 | No |
| 160 | ZC3H6 |  |  | 4681 | -0.021 | 0.1619 | No |
| 161 | BTBD3 |  |  | 4891 | -0.029 | 0.1386 | No |
| 162 | NOL4L |  |  | 4897 | -0.029 | 0.1387 | No |
| 163 | ZMYM4 |  |  | 4984 | -0.032 | 0.1296 | No |
| 164 | TULP4 |  |  | 5011 | -0.033 | 0.1274 | No |
| 165 | ZC2HC1A |  |  | 5107 | -0.037 | 0.1173 | No |
| 166 | GSTA4 |  |  | 5179 | -0.039 | 0.1101 | No |
| 167 | RALGDS |  |  | 5182 | -0.039 | 0.1107 | No |
| 168 | SNAP47 |  |  | 5217 | -0.041 | 0.1078 | No |
| 169 | SIAH1 |  |  | 5236 | -0.042 | 0.1066 | No |
| 170 | C16orf45 |  |  | 5278 | -0.043 | 0.1029 | No |
| 171 | FBXO21 |  |  | 5379 | -0.048 | 0.0925 | No |
| 172 | ATXN7L3B |  |  | 5403 | -0.049 | 0.0910 | No |
| 173 | PDE4DIP |  |  | 5468 | -0.051 | 0.0848 | No |
| 174 | ARMCX1 |  |  | 5476 | -0.052 | 0.0852 | No |
| 175 | PRAF2 |  |  | 5478 | -0.052 | 0.0862 | No |
| 176 | MAPRE3 |  |  | 5485 | -0.052 | 0.0867 | No |
| 177 | KIAA0930 |  |  | 5487 | -0.052 | 0.0877 | No |
| 178 | APBB1 |  |  | 5504 | -0.053 | 0.0871 | No |
| 179 | PLEKHO1 |  |  | 5598 | -0.057 | 0.0777 | No |
| 180 | SENP6 |  |  | 5599 | -0.057 | 0.0790 | No |
| 181 | MIR4458HG |  |  | 5631 | -0.059 | 0.0768 | No |
| 182 | NUDT3 |  |  | 5649 | -0.060 | 0.0762 | No |
| 183 | TP53BP1 |  |  | 5676 | -0.061 | 0.0746 | No |
| 184 | TMOD2 |  |  | 5775 | -0.065 | 0.0648 | No |
| 185 | SHC1 |  |  | 5833 | -0.067 | 0.0598 | No |
| 186 | MTURN |  |  | 5839 | -0.067 | 0.0607 | No |
| 187 | CNRIP1 |  |  | 5897 | -0.069 | 0.0557 | No |
| 188 | KDM5B |  |  | 5937 | -0.071 | 0.0529 | No |
| 189 | MPP6 |  |  | 5947 | -0.072 | 0.0535 | No |
| 190 | EPB41L1 |  |  | 5966 | -0.072 | 0.0530 | No |
| 191 | CEP170 |  |  | 5994 | -0.074 | 0.0516 | No |
| 192 | TAOK1 |  |  | 6046 | -0.077 | 0.0475 | No |
| 193 | TBCD |  |  | 6215 | -0.086 | 0.0301 | No |
| 194 | SMARCA1 |  |  | 6218 | -0.086 | 0.0318 | No |
| 195 | FOXO3 |  |  | 6238 | -0.087 | 0.0316 | No |
| 196 | HIPK2 |  |  | 6320 | -0.091 | 0.0244 | No |
| 197 | FNBP1L |  |  | 6401 | -0.096 | 0.0174 | No |
| 198 | HDGFL3 |  |  | 6415 | -0.096 | 0.0180 | No |
| 199 | THRA |  |  | 6448 | -0.098 | 0.0166 | No |
| 200 | CDC42EP4 |  |  | 6451 | -0.099 | 0.0186 | No |
| 201 | EIF2AK2 |  |  | 6481 | -0.100 | 0.0175 | No |
| 202 | CCSER2 |  |  | 6527 | -0.103 | 0.0146 | No |
| 203 | IFT20 |  |  | 6550 | -0.105 | 0.0145 | No |
| 204 | PBX1 |  |  | 6551 | -0.105 | 0.0168 | No |
| 205 | RAB40B |  |  | 6574 | -0.106 | 0.0167 | No |
| 206 | HIST3H2A |  |  | 6591 | -0.108 | 0.0173 | No |
| 207 | C5orf24 |  |  | 6603 | -0.108 | 0.0185 | No |
| 208 | ENAH |  |  | 6658 | -0.111 | 0.0148 | No |
| 209 | SMAD1 |  |  | 6662 | -0.112 | 0.0169 | No |
| 210 | SPIN1 |  |  | 6681 | -0.113 | 0.0174 | No |
| 211 | PJA2 |  |  | 6820 | -0.122 | 0.0043 | No |
| 212 | GNG2 |  |  | 6841 | -0.124 | 0.0048 | No |
| 213 | BRD3 |  |  | 6844 | -0.124 | 0.0074 | No |
| 214 | NEDD4L |  |  | 6876 | -0.128 | 0.0067 | No |
| 215 | SESN3 |  |  | 6935 | -0.132 | 0.0030 | No |
| 216 | ZEB1 |  |  | 6944 | -0.132 | 0.0050 | No |
| 217 | STX2 |  |  | 6946 | -0.132 | 0.0079 | No |
| 218 | LINC00667 |  |  | 6988 | -0.136 | 0.0062 | No |
| 219 | VEZF1 |  |  | 7040 | -0.140 | 0.0035 | No |
| 220 | PRKACB |  |  | 7086 | -0.144 | 0.0016 | No |
| 221 | PTPRN2 |  |  | 7125 | -0.148 | 0.0006 | No |
| 222 | USP11 |  |  | 7153 | -0.151 | 0.0009 | No |
| 223 | RPL7L1 |  |  | 7189 | -0.154 | 0.0003 | No |
| 224 | FKBP1B |  |  | 7211 | -0.156 | 0.0014 | No |
| 225 | CRK |  |  | 7218 | -0.158 | 0.0043 | No |
| 226 | PCGF2 |  |  | 7272 | -0.164 | 0.0019 | No |
| 227 | TSPYL1 |  |  | 7312 | -0.168 | 0.0012 | No |
| 228 | ZNRF1 |  |  | 7380 | -0.179 | -0.0025 | No |
| 229 | GSTM3 |  |  | 7412 | -0.183 | -0.0019 | No |
| 230 | SCRN1 |  |  | 7434 | -0.185 | -0.0002 | No |
| 231 | PPP2R2B |  |  | 7511 | -0.192 | -0.0046 | No |
| 232 | HPCAL1 |  |  | 7544 | -0.196 | -0.0039 | No |
| 233 | IFI27L2 |  |  | 7612 | -0.206 | -0.0069 | No |
| 234 | PCBP4 |  |  | 7643 | -0.210 | -0.0057 | No |
| 235 | CPNE2 |  |  | 7784 | -0.231 | -0.0165 | No |
| 236 | DEAF1 |  |  | 7828 | -0.241 | -0.0161 | No |
| 237 | TRIM37 |  |  | 7850 | -0.243 | -0.0130 | No |
| 238 | MAP1LC3A |  |  | 7856 | -0.245 | -0.0081 | No |
| 239 | LARP6 |  |  | 7915 | -0.259 | -0.0089 | No |
| 240 | TCEA2 |  |  | 8114 | -0.299 | -0.0249 | No |
| 241 | DPYSL2 |  |  | 8121 | -0.301 | -0.0189 | No |
| 242 | VAT1 |  |  | 8221 | -0.331 | -0.0228 | No |
| 243 | PPP2CB |  |  | 8347 | -0.369 | -0.0288 | No |
| 244 | NR2F6 |  |  | 8401 | -0.388 | -0.0262 | No |
| 245 | CXXC5 |  |  | 8526 | -0.446 | -0.0304 | No |
| 246 | PCBD1 |  |  | 8533 | -0.448 | -0.0210 | No |
| 247 | PFN2 |  |  | 8545 | -0.455 | -0.0121 | No |
| 248 | UBE2E3 |  |  | 8594 | -0.484 | -0.0067 | No |
| 249 | MARCKS |  |  | 8667 | -0.537 | -0.0029 | No |
| 250 | BRI3 |  |  | 8800 | -0.651 | -0.0035 | No |
| 251 | TMSB10 |  |  | 8979 | -1.090 | 0.0006 | No |
Table: GSEA details [plain text format]

  

Fig 2: NB\_KI\_CLUSTER2: Random ES distribution      
 Gene set null distribution of ES for **NB\_KI\_CLUSTER2**
